# Liposomal and Nanostructured Lipid Nanoformulations of a Pentacyclic Triterpenoid Birch Bark Extract: Structural Characterization and In Vitro Effects on Melanoma B16-F10 and Walker 256 Tumor Cells Apoptosis

**DOI:** 10.3390/ph17121630

**Published:** 2024-12-04

**Authors:** Dumitriţa Rugină, Mihai Adrian Socaciu, Madalina Nistor, Zorita Diaconeasa, Mihai Cenariu, Flaviu Alexandru Tabaran, Carmen Socaciu

**Affiliations:** 1 Faculty of Veterinary Medicine, University of Agricultural Sciences and Veterinary Medicine, 400372 Cluj-Napoca, Romania; dumitrita.rugina@usamvcluj.ro (D.R.); mihai.cenariu@usamvcluj.ro (M.C.); alexandru.tabaran@usamvcluj.ro (F.A.T.); 2 Faculty of Medicine, University of Medicine and Pharmacy “Iuliu Hatieganu”, 400347 Cluj-Napoca, Romania; mihai.socaciu@umfcluj.ro; 3 Department of Biotechnology, BIODIATECH—Proplanta Research Centre for Applied Biotechnology in Diagnosis and Molecular Therapy, 400478 Cluj-Napoca, Romania; 4 Faculty of Food Science and Technology, University of Agricultural Sciences and Veterinary Medicine, 400372 Cluj-Napoca, Romania; nistor.madalina@usamvcluj.ro (M.N.); zorita.sconta@usamvcluj.ro (Z.D.)

**Keywords:** pentacyclic triterpenoids, PEGylated liposomes, nanolipid complexes, diffraction light scattering, melanoma B16-F10 and walker 256 carcinoma cells, cell cycle arrest, apoptosis

## Abstract

**Background/Objectives**: Pentacyclic triterpenoids are increasingly studied as anticancer agents with many advantages compared to synthetic chemotherapeutics. The aim of this study was to prepare liposomal and nanostructured lipid formulations including a standardized extract of silver birch (*Betula pendula*) outer bark (TTs) and to evaluate their potential as anticancer agents in vitro, using Melanoma B16-F10 and Walker carcinoma cells. **Methods**: Appropriate solvents were selected for efficient TTs extraction, and original recipes were used to obtain Pegylated liposomes and nanolipid complexes with entrapped TTs, comparative to pure standards (betulinic acid and doxorubicin) in similar conditions. The composition, morphology, and sizes of all nanoformulations were checked by high-performance liquid chromatography/mass spectrometry, Transmission Electronic Microscopy, and Diffraction Light Scattering. The entrapment efficiency and its impact on cell viability, cell cycle arrest, and apoptosis by flow cytometry was also measured on both cancer cell lines. **Conclusions:** The standardized TTs, including betulin, lupeol, and betulinic acid, showed good stability and superior activity compared to pure betulinic acid. According to experimental data, TTs showed good entrapment in liposomal and NLC nanoformulations, both delivery systems including natural, biodegradable ingredients and enhanced bioavailability. The apoptosis and necrosis effects were more pronounced for TTs liposomal formulations in both types of cancer cells, with lower cytotoxicity compared to Doxorubicin, and can be correlated with their increased bioavailability.

## 1. Introduction

**Figure 1:**
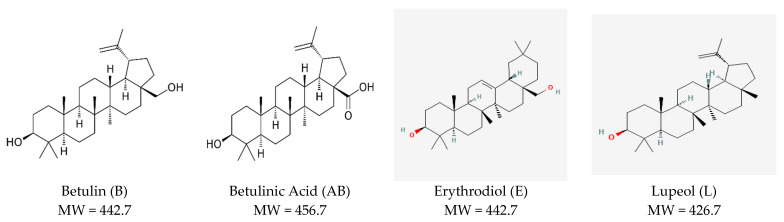
Most representative pentacyclic lupane-type triterpenoids in birch bark extracts: structure, molecular weight [MW], and maximal UV absorption.

Pentacyclic triterpenoids [TTs] represent a unique family of phytochemicals having a 30-carbon skeleton with five interconnected rings. It includes hydrocarbons (terpenes) and hydroxylated or carboxylated derivatives. Depending on their molecular structure, TTs are divided into five classes, the most known being lupane-type (lupeol, betulin, and betulinic acid) (Figure 1), oleanane-type (oleanolic acid, erythrodiol), and ursane-type (ursolic acid, uvaol).

One of the richest sources of pentacyclic triterpenoids is represented by birch (*Betula* species). In Central and Eastern Europe, the silver birch (*Betula Pendula Roth*) is dominant, and its outer bark contains betulin (B) as a major component (5–22%) and lupeol (approx. 1.3%), along with minor amounts of betulinic acid (AB) and traces of other pentacyclic triterpenes. The identification and isolation of these molecules, their bioavailability, and their applications were largely documented [1,2,3,4,5]. Their major disadvantage is the low solubility, and bioavailability, mainly due to their self-assembling with 3D nanosized structures, as demonstrated for ursolic acid [6].

The scientific interest in these molecules significantly increased after 1995 when B and AB demonstrated cytotoxicity against human Melanoma in vitro and in vivo [7,8,9]. Lupeol also proved to have cytotoxicity affecting the cellular viability [10]. Therefore, TTs as natural sources of medication have a powerful biological potential including antioxidants, anti-inflammatory, antiviral, and hypoglycemic, and especially as potent anticancer molecules, assuming their action as pharmacological agents [11,12,13,14,15]. Many publications were related to their isolation and cytotoxic effects, including patents and patent applications [4,8,16,17]. Non-malignant cells and tissues are not affected by AB, since it exerts its effects directly on the mitochondrion and triggers death selectively against cancerous cells, circumventing drug resistance in human cancers [18,19]. AB was initially considered a Melanoma-specific cytotoxic compound, inducing surface blebbing and cytoplasmic shrinking, and DNA fragmentation as indicative of the induction of apoptosis [7], but further evidence indicated a broader spectrum of activity against other cancer cell types, e.g., human neuroblastoma cell lines acting through induction of apoptosis independent of the p53 status [20], activation of caspases, production of reactive oxygen species, and permeability of transition pore openings [21,22]. Betulin was tested in vitro on A431, HeLa, and MCF7 cell lines, acting as an angiogenic inhibitor in vivo [23].

However, their hydrophobicity, poor permeability, and absorption lower their bioavailability and hinder their efficacy. Therefore, to enhance their efficacy by parenteral or skin administration, different non-toxic carriers were tested in recent decades. Nanoparticles and nanocolloids are increasingly used for terpenoid derivatives’ delivery in experimental in vitro and in vivo systems due to their multiple advantages, as reviewed [16,24,25,26,27,28,29,30,31].

Compared to conventional drugs, lipid-based nanoparticles that incorporate anticancer drugs have specific advantages, such as improved stability, permeability and retention, biocompatibility, and more precise targeting. These include liposomes, solid lipid nanoparticles (SLNs), nanostructured lipid carriers (NLCs), nanoemulsions, phytosomes, or nano-lipoproteins, whose preparation, characterization, and application in different drug delivery systems are continuously updated [32,33,34,35]. The most known nano-delivery vehicles are liposomes, especially used to incorporate hydrophilic drugs in the aqueous nucleus but also lipophilic drugs to be inserted in the membrane bilayer [36,37]. A better alternative for improved stability is the Polyethylene Glycol-modified (PEGylated) stealth liposomes applied on lipophilic anticancer agents. A drug release study showed that PEGylated AB liposomes of 142 nm had a better drug release effect than AB liposomes and better tumor inhibition compared to free AB or AB liposomes by in vitro and in vivo experiments [38].

Doxorubicin (DOXO) is an anthracycline antibiotic currently used in clinics as a hydrochlorinated formula. As an efficient anticancer drug, but one that is highly cytotoxic, it acts through topoisomerase I and II inhibition, mitochondrial apoptosis, caspases’ activation, production of reactive oxygen species, and anti-angiogenesis in cancer cells [11,39]. Liposomal DOXO proved high efficiency after encapsulation, with significant anticancer activity and reduced cardiotoxicity compared to free DOXO [36,40]. New versions of PEGylated liposomal DOXO were produced and improved its molecular targeting and tumor recognition [41,42,43], commercially known as Doxil^®^ or Caelyx^®^.

The development of lipid nanoparticles is one of the emerging applications in drug delivery, due to their unique composition- and size-dependent properties, as well as their being physiologically like body lipids, bioavailable and well tolerated. Nanostructured lipid carriers (NLCs) represent a second generation of SLNs [44,45,46], with many advantages over SLNs, by an enhanced drug loading capacity, improved stability, and different applications in both the pharmaceutical and cosmetic industries [34,47]. Different procedures were elaborated to obtain NLCs, but the most applied is the melt-emulsification and ultrasonication method, as recently reviewed [47,48]. The development of B-loaded NLCs (of around 200 nm) using emulsification was recently reported, showing their efficacy on Imiquimod-induced Psoriasis with an entrapment efficiency of 47 to 88% [49].

Our group has previous experience in preparing and characterizing birch extracts rich in TTs [2,16], as well as building liposomes and NLCs, which may incorporate different bioactive molecules [48,49,50,51]. The incorporation of TTs (B, AB, and L) in such carriers is still a challenge, as shown above. Their use for improved cell delivery and study of apoptotic effects comparatively represents not only scientific bottlenecks and starting points for innovative ways to deliver parenterally such anticancer molecules.

In this context, this study aims to apply cheap and easy procedures to obtain a bioactive extract rich in TTs, with a well-defined composition, and to incorporate it into PEGylated stealth liposomes (Lipo) and NLCs, compared with pure AB and Doxorubicin (as a positive control) in similar nanoformulations. Their size and structure as well as their effects on two cancer cell lines (Melanoma B16-F10 and Walker 256 carcinoma cells) were tested at different exposure times, as well as their impact on cell cycle and apoptosis, according to flow cytometry measurements.

## 2. Results

### 2.1. Preparation and Characterization of the TTs Extract

The TT extract was obtained in three steps by successive extractions of different solvents, as described in Section 4. The structure and solubility of TTs compared to pure AB in three different organic solvents were first checked and visualized by fluorescence microscopy, as shown in Figure S1 (Supplementary Files). Comparative to iso-propanol and ethanol/water mix, the mix of ethanol and DMSO (3:1) showed the best solubilization of TTs, resulting in a stable suspension.

The TTs extract was characterized first by UHPLC-QTOF-ESI^+^-MS analysis and four major pentacyclic terpenoids were identified and quantified, namely erythrodiol (E), betulin(B), betulinic acid (AB), and lupeol (L). Their identification was made by mass spectrometry, based on specific precursor ions and specific fragmentation, as shown in Figure S2 (Supplementary Files). The concentration of each component was expressed in AB equivalents (mg/mL) as described in the Materials and Methods. The total concentration of TTs in ethanol/DMSO (3:1) was 10.8 mg/mL AB eq. and the percentage of each component was found to be E: B: AB: L, 15.2:44.4:23.9:16.5 (w:w:w:w). This standardized extract (TTs) was used to build all nanoformulations and compared with similar formulations containing pure AB and Doxorubicin (as a positive control for anticancer effects).

### 2.2. Preparation and Characterization of the Nanoformulations with Entrapped TTs, AB and Doxo

**Figure 2:**
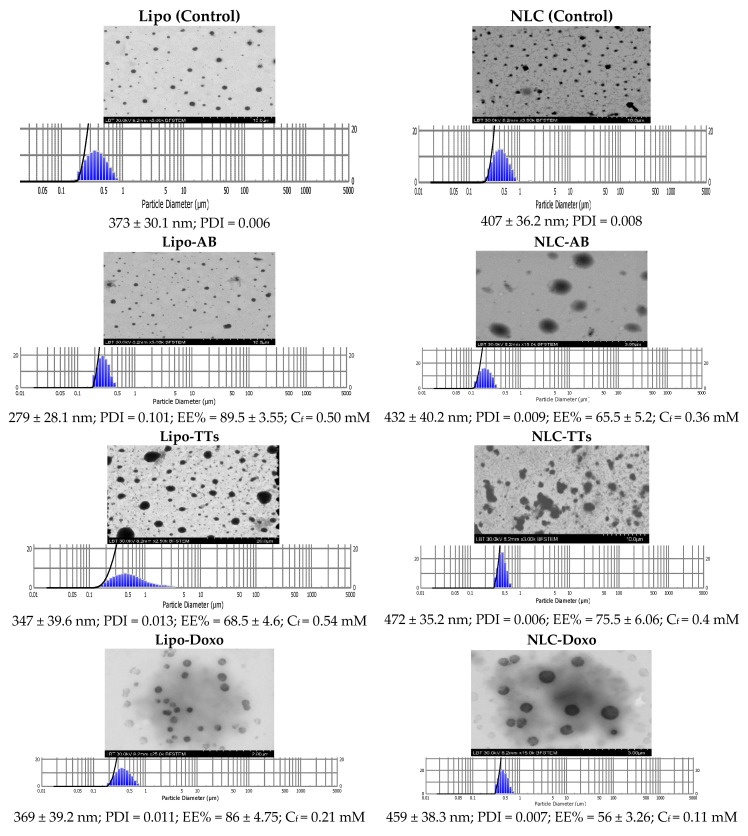
Morphology of Lipo and NLC nanoformulations containing AB, TTs, and Doxo, comparative to controls, as determined by TEM. Size distribution diameters (mean ± SD) (nm) of the formulations, the encapsulation efficiency (EE% ± SD), and the PDI values are included. The final concentrations (C_f_) for each nanoformulation including AB, TT, and Doxo were mentioned.

The images of the different variants of Lipo- and NLC-nanoformulations are presented in Figure S3 (Supplementary Files). The morphology of Lipo- and NLC-nanoformulations containing AB, TTs, and Doxo, comparative to controls, as determined by TEM, is presented in Figure 2. The figure includes also the size distribution and diameters (mean ± SD (nm) of all formulations, the encapsulation efficiency (EE% ± SD), and the PDI values. The final concentrations (Cf) of AB, TTs, and Doxo after entrapment were calculated according to the EE values, as described in Section 4 with Materials and Methods.

As shown, the population of nanoparticles had spheric shapes, and sizes between 279 and 472 nm, the NLCs being bigger, but not significantly, and showed to be inserted in a cloudy network, with increased tendency for aggregation compared to Lipo-structures. The Polydispersibility Index (PDI) showed a good distribution of nanoparticles in all cases. The stability of both types of formulations was high, with constant sizes, as checked, for one month of storage at 4 °C. Significant correlations were found between microscopic evaluation and the DLS measurements, as shown above. The encapsulation efficiency was higher in Lipo-formulations for AB and Doxo, while TTs (including more unpolar molecules) showed a higher encapsulation efficiency in NLC formulations.

### 2.3. Cell Viability and Cytotoxicity

The viability of both cell lines indicated, in general, dose-dependent and time-dependent decreases in viability. In general, the cytotoxic effects were more pronounced in the range of 4–24 h and were more pronounced in the Walker cell line. Figure S4 (Supplementary Files) includes the graphs representing the viability of Walker 256 cancer cells after the incubation (4, 24, 48 h) with successive concentrations (0.2–30 µM) of Lipo-AB, Lipo-TT, NLC-AB, and NLC-TT comparative to controls (Lipo and NLC). The graphs for Doxo, Lipo-Doxo, and NLC-Doxo are available from our previous publication [51]. Table 1 includes the comparative IC50 values (µM) of Doxo, AB, and TT delivered from Lipo- and NLC-formulations vs. free Doxo, AB, TT, and controls (Lipo- or NLC) in Melanoma B16-F10 and Walker 256 cell cultures, after 24 h of incubation.

In all cases, a more significant decrease in viability was noticed from 4 to 24 h. Table 1 presents the IC50 values (µM) of Doxo, AB, and TTs, delivered from Lipo- and NLC versus free forms (DOXO, AB, TTs) in Melanoma B16-F10 and Walker 256 cell cultures, after 24 h of incubation. The statistical significance of differences between Melanoma B16-F10 and Walker 256 cell, as well as between the different formulations, was mentioned also.

The controls (Lipo- and NLC) did not show a significant cytotoxic effect; nevertheless, NLC affected the cell viability of around 10% in both cell lines. Free Doxo showed higher cytotoxicity in Melanoma vs. Walker cell lines, with the mean IC_50_ values ranging from 0.18 to 0.87 µM, while, after entrapment, these values increased to 1.03 to 1.66 µM in Lipo- and 2.14–1.35 µM in NLC. According to statistical significance (Table 1B), the Doxo-nanoformulations were less toxic compared to free Doxo. Lipo-AB and NLC-AB showed higher IC_50_ values, especially in Melanoma cells. Comparatively, Lipo-TTs at similar concentrations proved to be less toxic in both cell lines, while NLC-TTs affected more Walker cell viability compared to free TTs (*p* < 0.01).

To conclude, the viability/cytotoxicity data according to IC_50_ values showed that Melanoma cells were more affected than Walker cells when free molecules were delivered to cells, and a gradual decrease in toxicity was observed in most cases when molecules were inserted in nanoformulations. When comparing AB and TTs nanoformulations, Lipo-AB were more toxic than NLC-AB, while NLC formulations of TTs were more toxic than Lipo-TTs. These data can be explained by a higher solubility and bioavailability of AB in liposomal formulations versus a higher solubility and bioavailability of TTs in NLC formulations.

### 2.4. Cell Cycle Distribution

**Figure 3:**
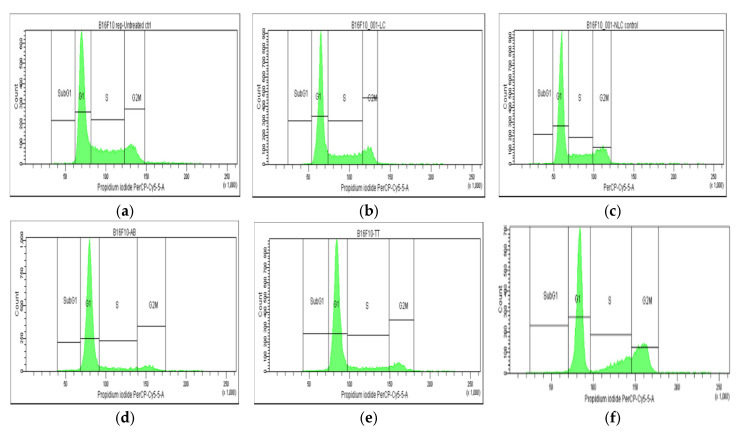

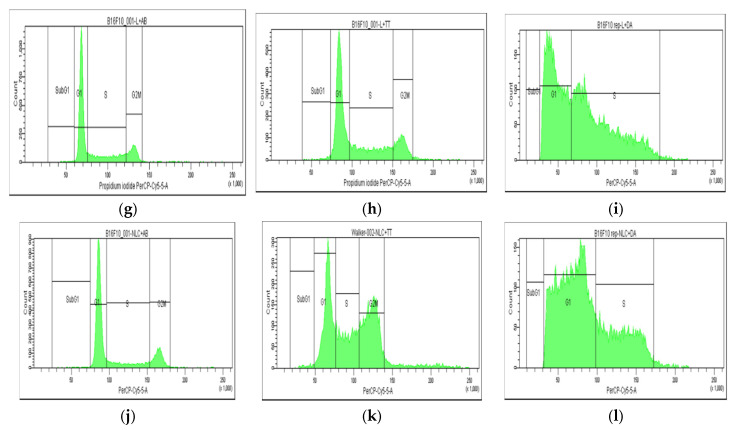
(**a**–**l**) The flow cytometry distribution of cell cycle affected in Melanoma B16-F10 cell population. (**a**) Non-treated cells. (**b**,**c**) After incubation with Lipo and NLC controls. (**d**–**f**) After incubation with AB, TTs, and Doxo. (**g**–**i**) After incubation with Lipo-AB, Lipo-TTs and Lipo-Doxo. (**j**–**l**) After incubation with NLC-AB, NLC-TTs, NLC-Doxo.

**Figure 4:**
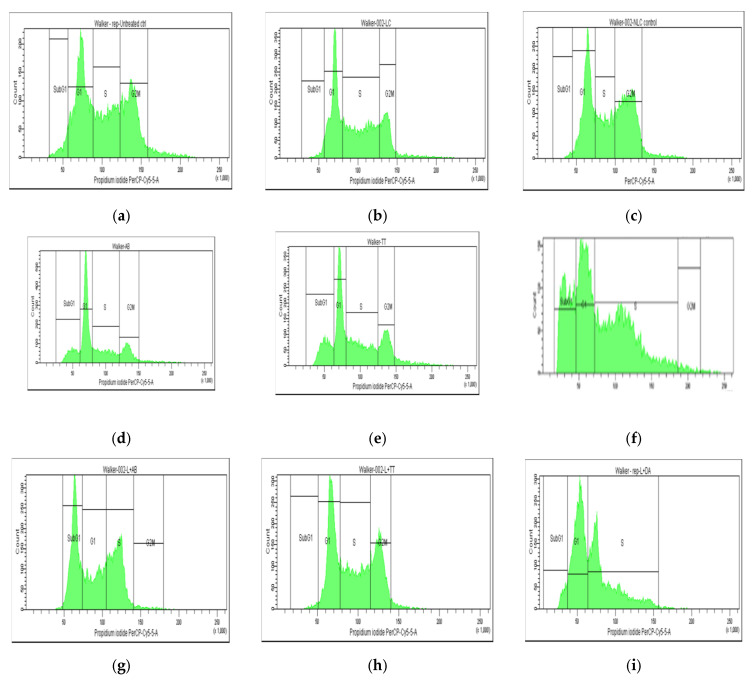

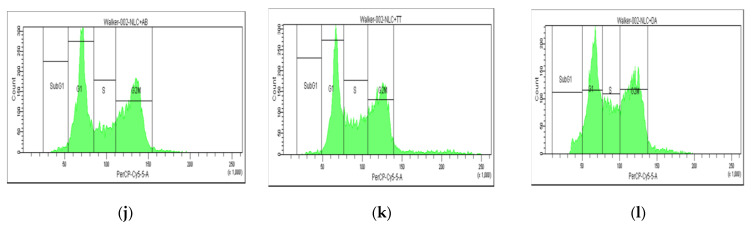
(**a**–**l**) The flow cytometry distribution of cell cycle affected Walker 256 cacinoma cell population. (**a**) Non-treated cells. (**b**,**c**) After incubation with Lipo and NLC controls. (**d**–**f**) After incubation with AB, TTs, and Doxo. (**g**–**i**) After incubation with Lipo-AB, Lipo-TTs and Lipo-Doxo. (**j**–**l**) After incubation with NLC-AB, NLC-TTs, and NLC-Doxo.

The results of cell cycle analysis performed for the two cell cultures (Melanoma B16-F10 and Walker 256 cells) using flow cytometry are shown in Figure 3 and Figure 4. The concentrations of Doxo, AB, and TTs as free or in nanoformulations corresponded in each case to the IC_50_ registered at 24 h cultivation for viability test (as presented in Table 1A). The percentage of Melanoma cell population affected (by counting and distribution) and inhibition of specific cell cycle stages (subG1, G1, G2, and S phases) were recorded and presented below for AB, TTs, Doxo, and each Lipo- and NLC-formulation (Figure 3d–l), comparative to control cell culture and free Lipo- and NLC- (Figure 3a–c).

**Figure 5:**
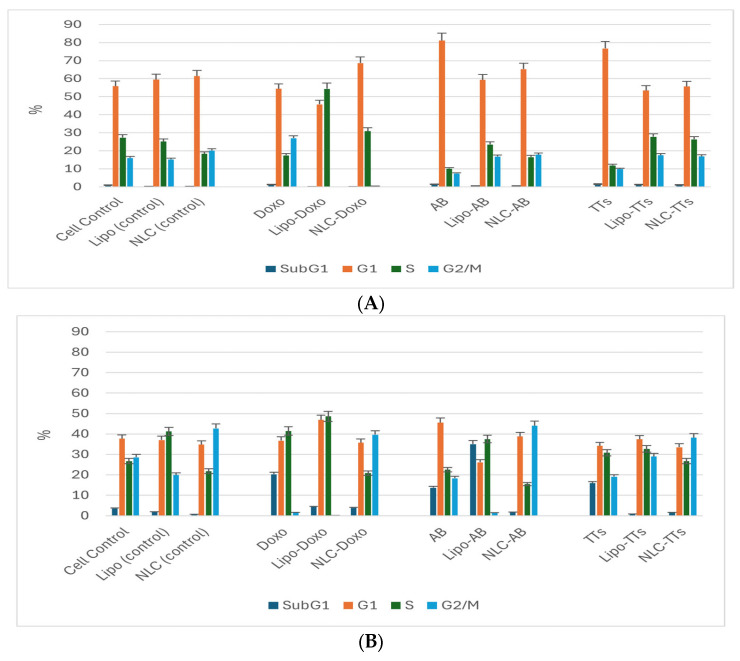
Comparative effects of Lipo- and NLC-nanoformulations and free extracts (TTs, AB and Doxo) on Melanoma B16-F10 (**A**) and Walker 256 (**B**) cell cycle.

The percentages of cell populations arrested in subG1, G1, G2, and S phases are shown as mean ± SD in Figure 5A,B.

No significant modifications were observed between the distribution of the Melanoma cell population (%) in non-treated controls compared to Lipo and NLc controls (Figure 3a–c and Figure 5A). The free AB and TTs showed similar effects with the highest counts in G1, while Doxo showed increased counts in S and especially G2M. The nanoformulas including AB or TTs showed similar effects, with significant arrests in the S and G2M phases. Lipo-AB and NLC-AB showed similar distribution profiles while Lipo-TTs and especially NLC-TTs showed significant arrests in S and G2M phases. 

On a similar scale, the percentage of the Walker cell population was differently distributed compared to Melanoma cells, with lower distribution in the G1 phase, due to a high rate of proliferation (in the S and G2M phases). In this case, Lipo and NLC controls affected the cell cycle differently, with Lipo- increasing the cell arrest in the S phase while NLC inducing the cell arrest in the G2M phase. The free Doxo, AB, and TTs induced cell arrests, especially in the subG1 phase compared to controls. Significant differences were noticed also between the nanoformulations with entrapped Doxo, AB, and TTs: Lipo-Doxo showed an increased cycle arrest in the S phase while NLC-Doxo induced the cell arrest in the G2M phase. Lipo-TTs induced more significant arrests in G2M while Lipo-AB was more in the subG1 phase. NLC-TTs and NLC-AB showed similar effects, with a higher percentage of arrested cells in the G2M phase. These data confirm the apoptotic effect of TTs, as well as AB, like Doxo, by shifting the cell cycle arrest to S and G2M phases, either as Lipo- or in NLC-formulationsTo confirm and detail the apoptotic mechanisms, a complementary evaluation was performed, as presented below.

### 2.5. Apoptosis

**Figure 6:**
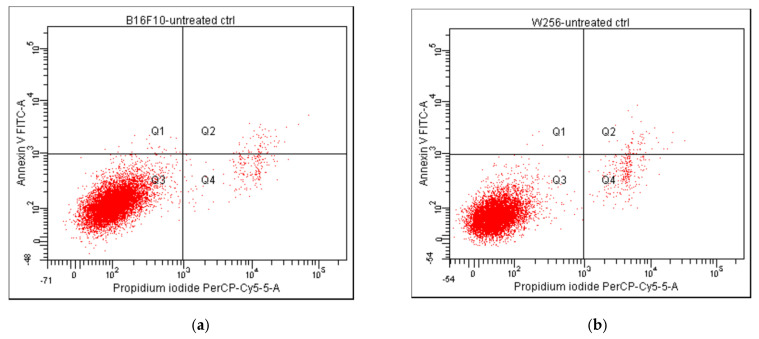

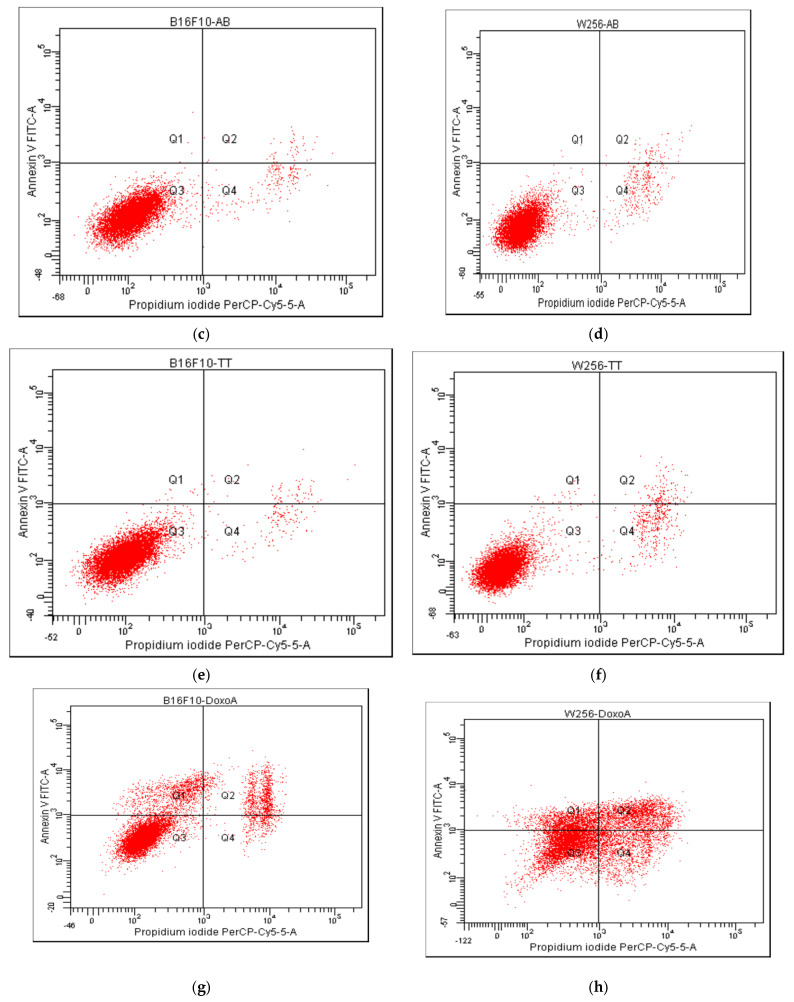
Comparative results of Annexin V/propidium iodide-stained flow cytometry for Melanoma B16F10 (**a**,**c**,**e**,**g**) and Walker 256 cells (**b**,**d**,**f**,**h**) after 24 h of incubation. (**a**) Melanoma B16F10 untreated cells; (**b**) Walker 256 untreated cells; (**c**,**e**,**g**) Melanoma B16F10 incubated with AB, TTs and Doxo; (**d**,**f**,**h**) Walker 256 cells incubated with AB, TTs, and Doxo. Q1 + Q2 = A (early and late apoptosis); Q3—viable cells; Q4—necrosis.

The progression of apoptosis was monitored also by flow cytometry, using the standard procedure with propidium iodide (PI) and fluorescein isothiocyanate (FITC)-conjugated Annexin V (Annexin V-FITC). The results are presented in Figure 6, comparatively showing the results for Melanoma B16F10 (a,c,e,g) and Walker 256 cells (b,d,f,h), as untreated cells (a,b) and after incubation for 24 hrs with Ab, TTs, and Doxo. According to the plots in quadrant Q1 (Annexin V-FITC+/PI−), the early apoptotic cells are marked, whereas, in quadrant Q2 (Annexin V-FITC+/PI+), the late apoptotic cells (end-stage, due to a loss of plasma membrane integrity) are distributed. The necrotic cells are in quadrant Q4, while viable cells (Annexin V-FITC−/PI−) are in quadrant Q3.

**Figure 7:**
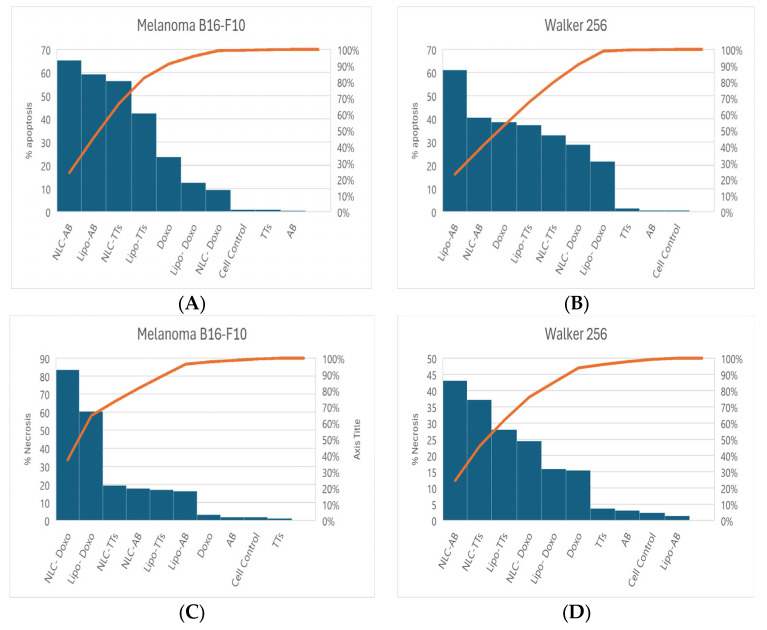
Histograms showing the comparative percentages of apoptotic cells ((**A**) melanoma B16-F10; (**B**) Walker 256) and necrotic cells ((**C**) melanoma B16-F10; (**D**) Walker 256) after the treatment with different Lipo- and NLC-formulations comparative to free molecules Doxo, AB, and TTs controls. A = Q1 + Q2 (early and late apoptosis); Q4—necrosis.

The non-treated cells (Figure 6a,b) showed a non-significant percentage of cells in apoptosis, while the strongest apoptotic and necrotic effects were noticed after the treatment with 0.87 µM Doxo, especially in Walker 256 cells (Figure 6g,h). The same cells treated with AB (10.22 µM in Melanoma and 13.53 µM in Walker cells) and TTs (19.25 µM in Melanoma and 28.53 µM in Walker cells) showed similar behavior and a lower impact on apoptosis compared to Doxo. The nanoformulations, at the same range of concentrations, showed significantly different impacts, as presented in Figure 7.

While Lipo-Doxo and NLC-Doxo at around 1 µM decreased the apoptotic effect compared to free Doxo, for the nanoformulations with entrapped triterpenoids (AB and TTs) at similar concentration ranges, from 14.42 to 27.33 µM and 19.48 to 27.25 µM, respectively, enhanced apoptotic effects were observed. Lipo-formulations of TTs showed similar apoptotic effects in both cancer cells, while NLC formulations were more active on Melanoma cells. Meanwhile, the necrotic effects were predominant in Doxo-entrapped nanoformulations, and superior to terpenoid nanoformulations. In Walker cells, the NLC formulations containing AB or TTs induced more necrosis than Lipo-formulations, in correlation with stronger cytotoxicity (see Table 1).

These data were positively correlated with the cell cycle measurements, as presented in Figure 3, Figure 4 and Figure 5, and confirmed by complementary measurements on caspase 3/7 activation.

## 3. Discussion

In the current study, an extract rich in pentacyclic terpenoids (TTs including betulin, AB, lupeol, and erythritol) from the outer bark of silver, was characterized and entrapped in two types of nanoformulations (liposomes and nanolipid complexes), to increase their bioavailability and activity as anticancer agents in two types of cancer cells: Melanoma B16-F10 and Walker 256 carcinoma cells. Different morphology parameters, sizes, and entrapment efficiencies were determined and compared with pure betulinic acid, the confirmed anticancer terpenoid, and Doxorubicin, the studied terpenoid, which proved the parameters, from including pure betulinic acid and Doxorubicin, considered the most effective anticancer drugs used in clinics.

The viability/cytotoxicity data according to IC_50_ values showed that Melanoma cells were more affected than Walker cells when free molecules were delivered to cells and a gradual decrease in toxicity was observed in most cases when molecules were inserted in nanoformulations. When compared to TTs and AB nanoformulations, Lipo-AB were more toxic than NLC-AB, while NLC formulations of TTs were more toxic than Lipo-TTs. These data can be explained by a higher solubility and bioavailability of AB in liposomal formulations *versus* a higher solubility and bioavailability of TTs in NLC formulations. Both types of nanoformulations enhanced active loading into both cancer cells with dose- and time-related cytotoxicity and improved bioavailability, enhancing the intracellular accumulation and their apoptotic and or necrotic effects.

We applied an improved version of PEGylated liposomes to entrap AB and TTs or Doxo according to previous findings [52,53], new recipes for NLCs, as well updated protocols for delivery systems recently documented [54,55,56]. These results confirm the complexity of cytotoxicity studies involving nanoparticles, where the cell cycle arrest varies significantly depending on the composition, size, and size distribution of formulations. In addition, the results vary depending on cellular types and experimental conditions, including the measurement protocols (e.g., flow cytometry, laser scanning cytometry) [57].

According to our experimental data, TT extract showed good entrapment in both liposomal and NLC nanoformulations, as these delivery systems have the advantage of including natural and biodegradable ingredients that can also be found in the vascular system and enhance bioavailability. The apoptosis and necrosis effect were more pronounced for liposomal formulations in both types of cancer cells and can be correlated with their increased bioavailability.

Complementary findings were obtained from in vivo experiments on Walker 256 rat carcinoma, where similar formulations were studied, and confirmed the apoptotic and necrotic effects of these terpenoid nanoformulations.

## 4. Materials and Methods

### 4.1. Extraction of TTs from Birch Bark

The bark of silver birch (*Betula pendula*) was collected in the Transylvania region in September 2023. The detached outer layer was dried for 24 h at 45 °C and stored in a dark desiccator. After grinding, 5 g of powder was washed 2× with petroleum ether to eliminate resins and then extracted two times with 100 mL mixture of iso-propanol/ethyl acetate, 1:1 (*v*/*v*), for 48 h at 40 °C. The extract was then evaporated under vacuum and re-extracted in iso-propanol, ethanol/water (1:1), or ethanol/dimethylsulfoxide (DMSO) 3:1. To evaluate the structural conformation, fluorescence microscopy was applied, using a trinocular digital Microscope IM-3LD4D (Optika, Italy) with green (emission/excitation 575/550 nm) and blue filters (emission/excitation 700/650 nm). Since the extract in ethanol/DMSO showed a stable colloid suspension, this type of extract was used in further experiments. Using high-performance liquid chromatography coupled with MS spectrometry (UHPLC-ESI+MS), the qualitative and quantitative composition of TTs extract was determined as described in Figure S2 (Supplementary Files) using a calibration curve with pure AB, in the range of 2–10 mg/mL. The composition of TTs included erythrodiol E (Rt = 9.38 min), betulin B (Rt = 9.9 min), betulinic aid AB (RT = 10.1 min) and lupeol L (10.6 min) in a total concentration of 10.8 mg/mL expressed in equivalents AB and the weight percentages of the four components were E:B:AB:L,15.2:44.4:23.9:16.5. This standardized extract was used in all experiments.

### 4.2. Preparation of PEGylated Liposomes Using the Ethanol Injection Method

The procedure applied was adapted from Fan et al, 2008 [53]. The lipid phase (LP) included 16 mL of soybean lecithin (Sigma Aldrich, Merck, Darmstadt, Germany) 6.25% and 25 mg of cholesterol dissolved in pure ethanol (HPLC grade, Merck, Darmstadt, Germany). The aqueous phase (AqP) included 40 mL of phosphate buffer, 0.01 M of pH 6.5, 40 µL of Tween-80, and 20 mg of PEG2000. The AqP was heated on a water bath and under magnetic stirring at 60 °C, and the LP heated at 65 °C was added dropwise for about 15 min, with a ratio of AqP/LP 2:1 (v:v). After a further mix for another 15 min, to evaporate the ethanol, the liposomal suspension was ultrasonicated at a high amplitude, using an UP50H Compact Lab Homogenizer (Hielscher, Teltow, Germany). With empty control liposomes and liposomes containing Doxorubicin (Doxo), AB and TT extracts were prepared using this procedure.

Preparation for Lipo-Doxo: An amount of 2 mg/mL (3.45 mM) of Doxorubicin hydrochloride (Doxo) saline solution was purchased as a commercial perfusion solution (Accord Healthcare Ltd., Newcastle, UK). This solution was diluted up to a final concentration of 2 mM, using a mix of ethanol/DMSO 3:1.A volume of 5 mL of Doxo (2 mM) was mixed with 32 mL of AqP and, following the procedure mentioned before, 16 mL of LP was added, and after ethanol evaporation, a volume of 40 mL of liposomal suspension was obtained with a theoretical concentration of 0.25 mM of Doxo. The entrapment rate is described below.

Preparation for Lipo-AB and Lipo-TTs: Following the same procedure, a 1 mL solution of ethanol/DMSO containing 10 mg/mL AB and 10.8 mg/mL TTs, respectively, was mixed with 15 mL of FL. Then, the liposomes were built similarly using a volume of 32 mL of AqP. The final suspension had 40 mL. The theoretical concentration of AB and TTs was (considering the MW of AB = 456.7 and mean MW for TTs = 443.5) 0.25 mg/mL and 0.27 mg/mL in each case, corresponding to 0.55 mM for AB and 0.6 mM for TTs. For all formulations, obtained in triplicate, the entrapment efficiency was determined as presented below.

### 4.3. Preparation of NLC Formulations

To obtain the NLC formulations, Compritol 888ATO, a standardized glycerol dibehenate (Gattefosse, Saint-Priest France) previously used in similar formulations [45,46], was mixed with stearic acid, oleic acid, Tween 80, and Triethanolamine, in a ratio of 10:5:2.5:2.5 (*w*/*w*). By hot emulsification procedure, a dropwise addition of 50 mL of hot ultrapure water phase (pH 7.2) to 2 g of melted lipid mix (at 80 °C) was applied using an ultraturax at 20,000 rpm speed for 15 min. The hot emulsion was ultrasonicated for 5 min with high amplitude using the UP50H Compact Lab Homogenizer (Hielscher). The resulting suspension (50 mL) suspension was kept on ice for 15 min. To obtain NLC-Doxo formulations, 5 mL of Doxo (2 mM) was included in 50 mL of the hot water phase, their theoretical concentration being 0.2 mM in the final suspension. Similarly, 1 mL solutions including 10 mg of AB and 15 mg of TTs in ethanol/DMSO (3:1) were introduced in 50 mL of hot water, their theoretical concentrations were considered to be 0.44 mM and 0.68 mM, respectively. For all formulations, obtained in triplicate, the entrapment efficiency was determined as presented below.

### 4.4. Entrapment Efficiency, Size Determination and Morphology of Liposomes vs. NLCs

To evaluate the percentage of Doxo, AB, and TTs entrapment in both systems (Lipo- and NLC-), Amicon^®^ Ultra 15 mL Centrifugal Filter devices (Merck Millipore, Darmstadt, Germany) with 100 K cut-off were used. The liposome or NLC suspensions were filtered by centrifugation at 4600 rpm for 30 min at 25 °C in a Hettich Rotofix 46 Centrifuge and the retentate was collected. The procedure was repeated two times. After restoration of the initial volume, the concentrations of Doxo, AB, and TTs in the retentate were determined in each case and compared to the theoretical concentration. The evaluation of entrapment efficiency (EE%) was made by UV-VIS spectrometry (Perkin Elmer Lambda 25, Waltham, MA, USA), recording the specific absorptions for Doxo (480 nm) and Ab or TT (210 nm) after dissolving the liposomal or NLC suspensions in a solution of 0.1% Triton X-100 in ethanol. The formula used to calculate was EE% = (At − Aret/At) × 100, where At represents the absorption of initial suspension; Aret represents the absorption of retentate suspension. The sizes of all formulations were determined by laser diffraction technique using the Shimadzu SALD 2300 DLS instrument (Shimadzu, Kyoto, Japan) with the software Wing SALDII version 3.4.10. The Polydispersibility Index (PDI) was also calculated in each case. The morphology and size were visualized in parallel by Transmission Electronic Microscopy (TEM-Hitachi SU8320 Krefeld, Germany) with a calibrated size range between 50 nm and 50 mm to obtain TEM images.

### 4.5. Cell Cultures

The Melanoma B16-F10 cell line, isolated from mouse skin tissue, was purchased from ATCC (Manassas, VA, USA) and cultured in growth media containing Dulbeco’s modified Minimum Essential Medium (DMEM) supplemented with fetal bovine serum (10%), L-glutamine (1%), and a mixture of penicillin–streptomycin (1%). Cells were maintained under standard conditions in a humidified atmosphere (95%) at 37 °C and 5% CO_2_. The Walker 256 cell line (synonym LLC-WRC 256) from a rat breast carcinoma, originally obtained from the American Type Culture Collection (ATCC) and adapted with an aggressive metastatic behavior in suspension (kindly donated by Prof. Oliver Thews, Martin Luther University, Halle-Wittenberg, Germany), was used as an appropriate model. This line was cultured in a Sigma RPMI-1640 growth medium containing 10% sterile fetal bovine serum, 20 mM of HEPES, 10 mM of L-glutamine, 10 mL of 7.5% NaHCO_3_, and 1 mL of penicillin and streptomycin (Sigma 10,000 units penicillin and 10 mg of streptomycin/mL) for each 100 mL volume. Both types of cell cultures were incubated in parallel with Doxo (0.5–3.5 µM), AB (0.5–30 µM), and TTs (0.5–30 µM) after dilution in ethanol/DMSO, 3:1 and addition to the cell cultures. The same concentrations were applied for the Lipo- and NLC-formulations.

### 4.6. Viability Assays

The cytotoxic impact of free molecules (AB, TTs) and Lipo- and NLC-formulations comparative to Lipo- and NLC-controls was measured using similar concentrations, in the range of 0–3.6 µM for Doxo, 0–30 µM for AB, and 0–25 µM for TTs. The viability was evaluated by the MTT assay applied on B16-F10 Melanoma cells and Walker 256 cells. Shortly, in a 96-well plate, 8 × 10^3^ B16-F10 cells and 1 × 10^4^ of Walker 256 cells were seeded. On the next day, cells were subjected to successive concentrations of free molecules and their formulations. At 4, 24, and 48 h after treatments, 10 µL of the 12 mM MTT solution in PBS was added in each well, followed by incubation at 37 °C for 4 h. Afterwards, 150 µL of a mixture of DMSO/SDS 30% (90:60) were added in each well, the plate was shaken for 5 min at room temperature, in the dark, and then incubated for 10 min at 37 °C. Using a microplate reader (Biotek, SynergyHT, Marshall Scientific, Hampton, NH, USA), the absorbance of the formazan was determined at 550 nm. Half-maximal inhibitory concentration (IC50) values, utilizing a sigmoidal fitting equation, were calculated (GraphPad Prism 10.1.2).

### 4.7. Cell Cycle Analysis by Flow Cytometry

For each cell line, 1 × 10^6^ cells were seeded in 6-well plates and left to adapt for 24 h (B16-F10 adherent cell line) and 2 h (Walker 256 cell line in suspension), respectively, prior to treatment. Separate sets of treatments were prepared for the two cell lines using the concentrations of all formulations corresponding to IC_50_ registered at 24 h cultivation for viability test. The cells were incubated for 24 h at 37 °C and, after the media removal (collected in a 15 mL tube), the adherent B16-F10 cells were detached with trypsin, their growth medium was also collected, and together were centrifuged for 3 min at 1500 rpm. The cellular pellet was washed 2× with cold PBS and fixed with 2–5 mL dropwise of 70% ethanol on ice. For the fixation procedure, both cell lines were kept at 4 °C for 30 min, and, then, the ethanol was removed by centrifugation (5 min, 2000 rpm), and a washing procedure with 2× PBS was performed. A volume of 50 μL RNAse (100 μg/mL) was added to all cell pellets, followed by an incubation at 37 °C for 15 min. Finally, 200 μL of propidium iodide (50 μg/mL), a nuclear staining dye was added for 10 min at 4 °C to measure the cell cycle. All data were collected, stored, and analysed using a BD FACS Canto II flux cytometer (Becton Dickinson, New York, NY, USA).

### 4.8. Apoptosis and Necrosis Assays by Flow Cytometry

The percentage of cells that undergo apoptosis and/or necrosis was measured by using the Annexin V–FITC and propidium iodide staining kit, provided by Merck (Darmstadt, Germany), and analyzed using the same BD FACS. The analysis was performed on each cell line: 1 × 10^6^ cells were seeded in 6-well plates, and left to adapt to the medium for 24 h (B16-F10), and 2 h (Walker 256), respectively, prior to treatment. Separate sets of treatments were prepared for each formulation. Next, the cells were incubated for 24 h at 37°. After the completion of the treatment time, the media was removed, and the cells were prepared for staining by washing twice with PBS (3 min, 1500 rpm centrifugation), submersed in 500 µL of binding buffer, provided by the kit, and transferred into flow cytometry testing tubes. Finally, 5 µL of Annexin V FITC and 10 µL of propidium iodide solution were added to each sample tube. After 10 min of incubation at room temperature, protected from light, the cells were diluted 2× with cell wash solution. All data were collected, stored, and analyzed also by BD FACS Canto II flux cytometer (Becton Dickinson, NJ, USA).

### 4.9. Statistics

The estimation of the statistical differences in this study used one-way ANOVA followed by post hoc Tukey’s test provided by GraphPad prism package software (La Jolla, CA, USA). The viability data are presented as mean ± SD. Values of *p* ˂ 0.1, *p* < 0.01, and *p* < 0.001 counted as thresholds for significant differences.

## 5. Conclusions

The pentacyclic triterpenoid extract showed good stability and synergistic effects of the main components and was superior compared to pure betulinic acid. According to experimental data, this extract showed good entrapment in liposomal and NLC nanoformulations in both delivery systems, including natural, biodegradable ingredients, and enhanced bioavailability. The apoptosis and necrosis effects were more pronounced for its liposomal formulations in both types of cancer cells, with lower cytotoxicity compared to Doxorubicin, and can be correlated with their bioavailability. The added value of the four terpenoid components (betulin, betulinic acid, erythrodiol, and lupeol) in this extract was demonstrated by amplified effects of the TTs extract, compared to pure betulinic acid, either free or entrapped in bioavailable nanoformulations. These data may fill the existing literature, which confirms the potential of pentacyclic triterpenoids as natural anticancer agents, and the potential of nanoformulations to improve their cellular uptake, influence cytotoxicity, and induce apoptosis or necrosis, as main mechanisms involved in the modulation of tumor cell inhibition.

## Figures and Tables

**Table 1:** The IC_50_ (mean ± SD) values (µM) of Doxo, AB, and TTs, free or after entrapment in Lipo and NLC in Melanoma B16-F10 and Walker 256 cell cultures, after 24 h of incubation. The statistical significance of differences between Melanoma B16-F10 and Walker 256 cell (A), as well as between the different formulations (B), was mentioned also, by t and *p* values (* *p* < 0.1; ** *p* < 0.01; *** *p* < 0.001). NS—nonsignificant.

A.	Melanoma B16-F10	Walker 256	t (M vs. W256)	Significance
Doxo	0.18 ± 0.5	0.87 ± 0.14	−2.555	NS
Lipo-Doxo	1.03 ± 0.11	1.66 ± 0.18	−10.203	**
NLC-Doxo	2.14 ± 0.12	1.35 ± 0.12	11.401	**
AB	10.22 ± 4.03	13.53 ± 1.65	−3.087	*
Lipo-AB	15.52 ± 1.1	14.42 ± 1.13	0.620	NS
NLC-AB	22.58 ± 0.03	27.33 ± 2.8	−279.883	***
TTs	19.25 ± 2.33	28.55 ± 3.2	−8.074	*
Lipo-TTs	23.58 ± 0.03	27.25 ± 1.8	−215.883	***
NLC-TTs	20.22 ± 0.9	19.48 ± 1.55	−0.111	NS
**B.**	**Melanoma B16-F10**		**Walker 256**	
	**t**	**Significance**	**t**	**Significance**
NLC-Doxo/Doxo	28.083	**	6.86	*
Lipo-Doxo/Doxo	13.023	**	7.540556	*
NLC-Doxo/Lipo Doxo	16.078	**	−4.70083	NS
NLC-AB/AB	716.970	***	6.975	*
Lipo-AB/AB	4.402	*	−0.27168	NS
NLC-AB/Lipo-AB	410.733	***	6.93875	*
NLC-TTs/TTs	−0.444	NS	−11.8774	**
Lipo-TTs/TTs	250.253	***	−2.67361	NS
NLC-TTs/Lipo-TTs	−6.563	*	−10.5726	**

## Data Availability

Data is contained within the article or Supplementary Material.

## Supplementary Materials

The following supporting information can be downloaded at: https://www.mdpi.com/article/10.3390/ph17121630/s1, Figure S1: comparative structures of the betulinic acid (A–C) and the TTs extract (D–F) in different solvents: iso-propanol (A,D), ethanol/water 1:1 (B,E), and ethanol/DMSO, 3:1 (C,F), as determined by fluorescence microscopy. Green filters were used for images B, C, and D, while blue filters were used for images A, E, and F. The fluorescent Sudan III was added for an optimized image of the structures in iso-propanol (A,D). Figure S2: birch bark extract (TTs) composition, according to UHPLC-ESI^+^-MS analysis. Figure S3: liposomal (Lipo-) and NLC formulations containing Doxorubicin (Doxo), betulinic acid (AB), and triterpene extract (TTs). Figure S4: viability of Walker 256 cancer cells after the incubation (4, 24, 48 h) with successive concentrations (0.2–30 µM) of Lipo-AB, Lipo-TT, NLC-AB, and NLC-TT as well compared to controls (Lipo and NLC).
