# Supplementary material for: Liposomal and Nanostructured Lipid Nanoformulations of a Pentacyclic Triterpenoid Birch Bark Extract: Structural Characterization and In Vitro Effects on Melanoma B16-F10 and Walker 256 Tumor Cells Apoptosis
Source: Pharmaceuticals (Basel). 2024 Dec 4;17(12):1630. doi: 10.3390/ph17121630 (PMC11728790; doi:10.3390/ph17121630)

Figure S2. Birch bark extract (TTs) composition, according to UHPLC-ESI+-MS analysis

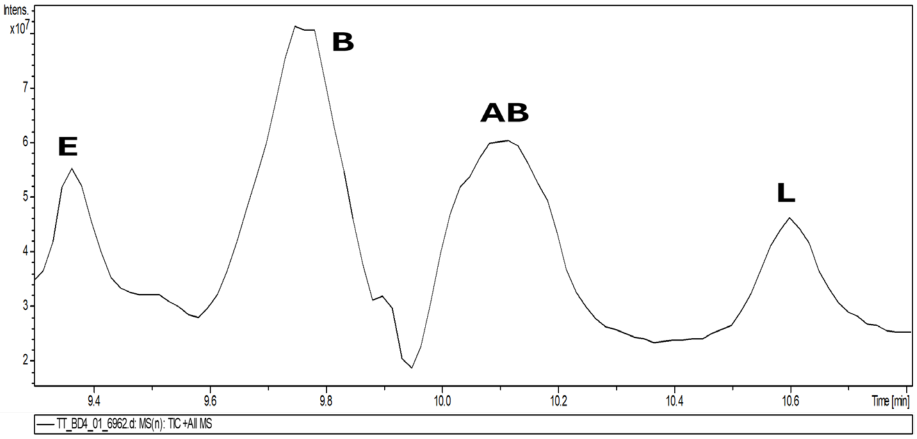

Total Ion Chromatogram

Compound	Rt(min)	Precursor [M+H]	[M+H-H_2O]	Other fragments
Erithrodiol (E)	9.38	443.3000	**425.1900**	
Betulin (B)	9.90	443.3066	**425.3495**	
Betulinic acid (AB)	10.10	**457.3238**	439.3279	
Lupeol (L)	10.60	427.2653	**409.2081**	**381.2760**

MS Spectrum View

E (Rt= 9.4 min)

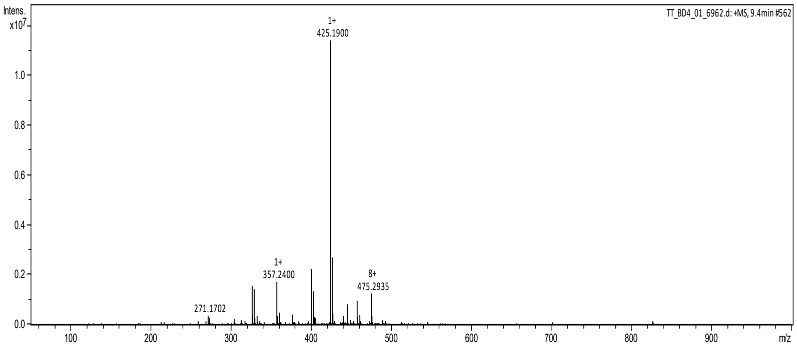

B (Rt= 9.9 min) in TTs and comparative with a pure standard

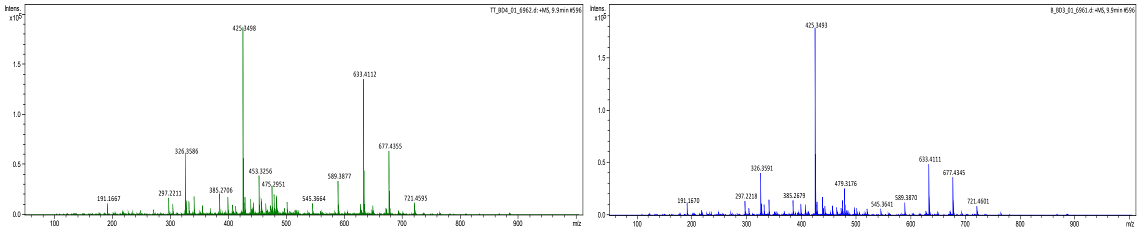

Betulin in TTs

Betulin -pure standard

AB (Rt= 10.1 min) in TTs and comparative with a pure standard

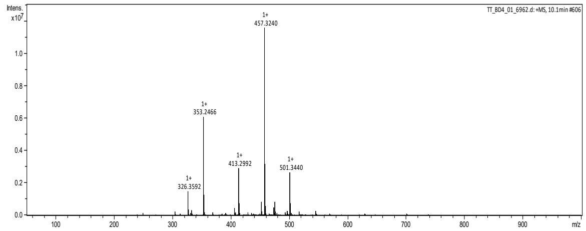

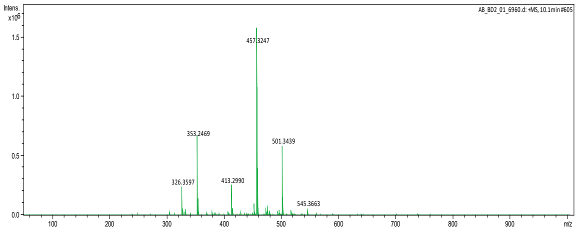

Betulinic acid in TTs

Betulinic acid -pure standard

L(Rt= 10.6 min) in TTs

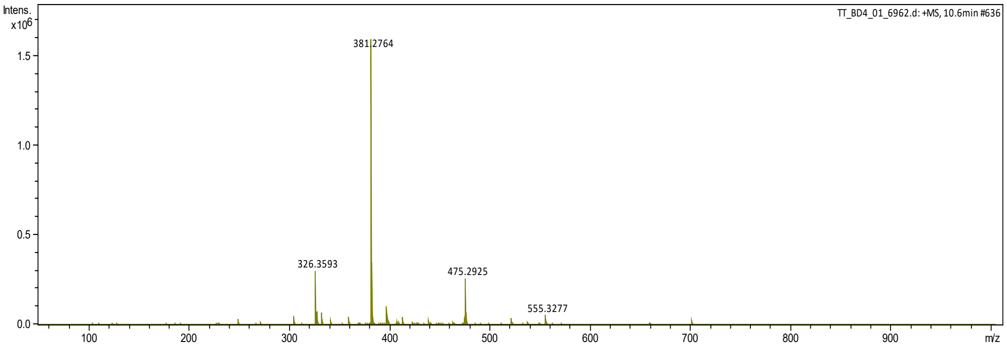

Supplement: Supplementary file 1 [file pharmaceuticals-17-01630-s001.zip › Figure S2. LC-MS data.pdf]
