# Supplementary material for: Liposomal and Nanostructured Lipid Nanoformulations of a Pentacyclic Triterpenoid Birch Bark Extract: Structural Characterization and In Vitro Effects on Melanoma B16-F10 and Walker 256 Tumor Cells Apoptosis
Source: Pharmaceuticals (Basel). 2024 Dec 4;17(12):1630. doi: 10.3390/ph17121630 (PMC11728790; doi:10.3390/ph17121630)
Supplement: Supplementary file 1 [file pharmaceuticals-17-01630-s001.zip › Figure S1. fluorescence microscopy.pdf]

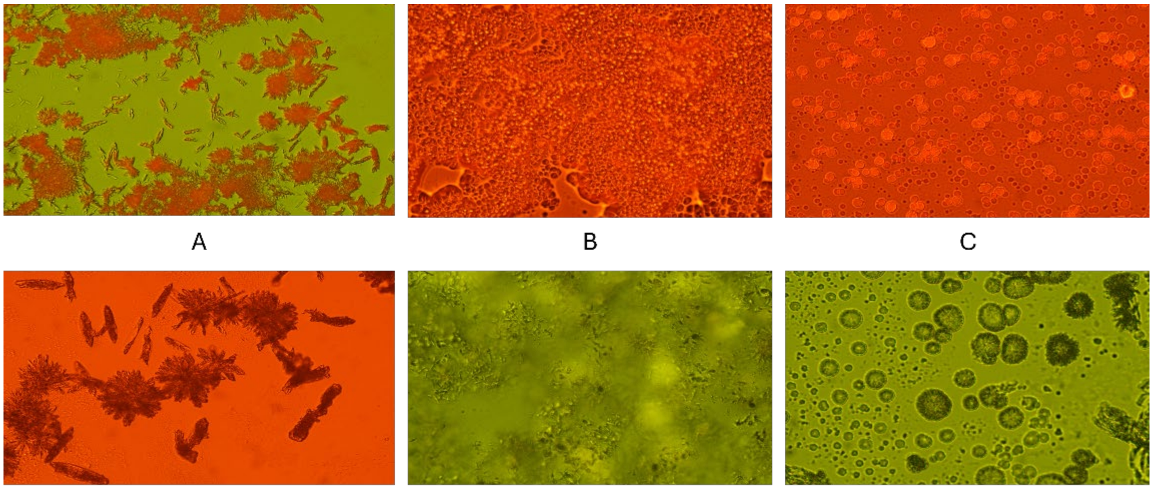

A B C

D E F

Figure S1. Comparative structures of the betulinic acid (A-C) and the TTs extract (D-F) in different solvents: iso-propanol (A and D), ethanol: water 1:1 (B and E) and ethanol: DMSO, 3:1 (C and F), as determined by fluorescence microscopy. Green filters were used for images B,C,D, while blue filters were used for images A,E,F. The fluorescent Sudan III was added for an optimized image of the structures in iso-propanol (A and D).
