# Supplementary material for: Liposomal and Nanostructured Lipid Nanoformulations of a Pentacyclic Triterpenoid Birch Bark Extract: Structural Characterization and In Vitro Effects on Melanoma B16-F10 and Walker 256 Tumor Cells Apoptosis
Source: Pharmaceuticals (Basel). 2024 Dec 4;17(12):1630. doi: 10.3390/ph17121630 (PMC11728790; doi:10.3390/ph17121630)
Supplement: Supplementary file 1 [file pharmaceuticals-17-01630-s001.zip › Figure S3 Images lipo and NLC.pdf]

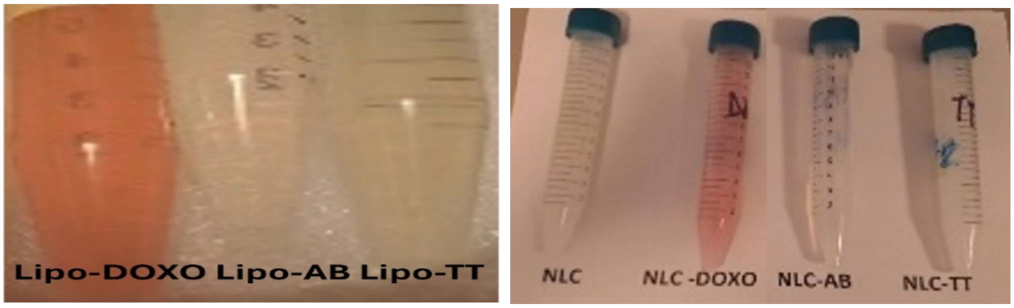

Figure S3. Liposomal (Lipo-) and NLC formulations containing Doxorubicin (DOXO), Betulinic acid (AB) and triterpene extract (TT).
