# Supplementary material for: Liposomal and Nanostructured Lipid Nanoformulations of a Pentacyclic Triterpenoid Birch Bark Extract: Structural Characterization and In Vitro Effects on Melanoma B16-F10 and Walker 256 Tumor Cells Apoptosis
Source: Pharmaceuticals (Basel). 2024 Dec 4;17(12):1630. doi: 10.3390/ph17121630 (PMC11728790; doi:10.3390/ph17121630)
Supplement: Supplementary file 1 [file pharmaceuticals-17-01630-s001.zip › Figure S4 Viability of Walker 256 cancer cells.pdf]

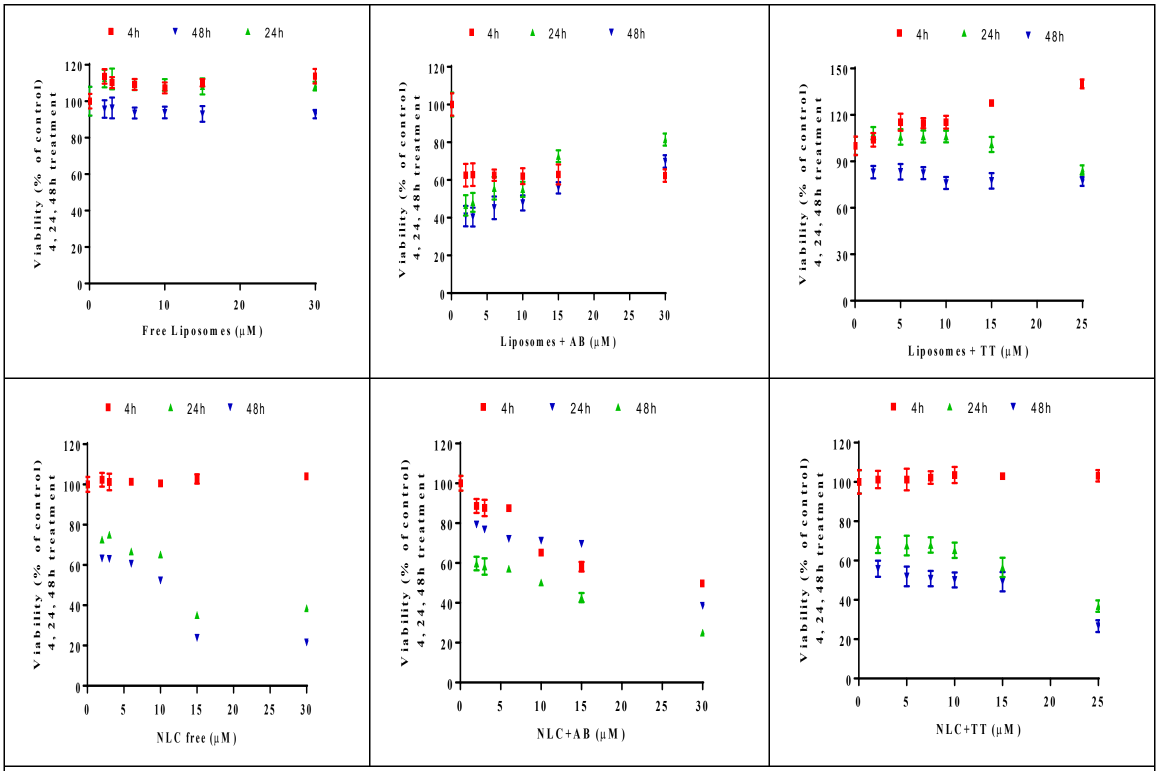

Figure S4. Viability of Walker 256 cancer cells after the incubation (4, 24, 48h) with successive concentrations (0.2-30 μM) of Lipo-AB, Lipo-TT, NLC-AB, NLC-TT comparative to controls (Lipo and NLC).
